# How Entrepreneurship Education at Universities Influences Entrepreneurial Intention: Mediating Effect Based on Entrepreneurial Competence

**DOI:** 10.3389/fpsyg.2021.655868

**Published:** 2021-07-06

**Authors:** Yijun Lv, Yingying Chen, Yimin Sha, Jing Wang, Lanyijie An, Tingjun Chen, Xiang Huang, Yangjie Huang, Leilei Huang

**Affiliations:** ^1^Institute of China Innovation and Entrepreneurship Education, Wenzhou Medical University, Wenzhou, China; ^2^College of Humanities, Zhejiang Normal University, Jinhua, China; ^3^School of Education, Peking University, Beijing, China

**Keywords:** entrepreneurship education, entrepreneurial competence, entrepreneurial intention, entrepreneurship teaching, entrepreneurship practice support, business plan competition

## Abstract

Research shows that entrepreneurial activities significantly promote economic development, which enhances the importance of the innovative entrepreneurial potential of college students. This study analyzes the effect of entrepreneurship education on entrepreneurial intention from the perspective of planned behavior theory. By examining the significant role of entrepreneurship education at colleges and universities on economic and social development, we established a conceptual model. To understand the relationship between entrepreneurship education and entrepreneurial intention, the hypotheses propose the intermediary role of entrepreneurial ability, and the study provides evidence from China the relationship between entrepreneurship education and entrepreneurial intention. Improving entrepreneurial intention and encouraging college students to establish businesses through entrepreneurship education in universities is crucial. This study proposes a hypothetical model of the relationship between entrepreneurial competence and entrepreneurial intention in entrepreneurship education at universities. Using a questionnaire survey of college students with practical experience in the Yangtze River Delta of China, the bootstrap method in the SPSS macro program process software verifies the hypotheses. The results show that entrepreneurial teaching, business plan competition, and entrepreneurial practice support positively affect entrepreneurial competence. In addition, entrepreneurial competence plays an intermediary role in the relationship between entrepreneurial teaching, business plan competition, entrepreneurship practice support, and entrepreneurial intention. Entrepreneurship education improves the ability to establish a business in the present and in entrepreneurial activities in the future. Entrepreneurial competence obtained through entrepreneurship education continuously affects entrepreneurial intention.

## Introduction

With increasing downward economic pressure, providing sustained impetus and new vitality for economic development is essential (Raposo and Paço, 2011). Entrepreneurial activities promote social and economic development in multiple ways, as they increase individuals' incomes, create more jobs, and stimulate the innovative vitality of society. The level of entrepreneurial activity depends on the number of entrepreneurs available, and the higher the number of entrepreneurs, the more active entrepreneurial activity will be (Gerba, 2012). A large number of literature on entrepreneurship recognizes the contribution of entrepreneurs to national economic growth and development (Gerba, 2012; Schoon and Duckworth, 2012). An increase in the number of entrepreneurs can help the country's economy by creating jobs and reducing unemployment (Nabi and Holden, 2013). So how can we increase the number of entrepreneurs? Some scholars believe that entrepreneurs can be created by cultivating entrepreneurial qualities such as entrepreneurial knowledge, attitudes, and skills through education and encouragement of creativity (Otache, 2019). Public policy planners and government agencies worldwide recognized entrepreneurship education as a means to encourage social innovation (Jones and Iredale, 2014). Entrepreneurship education provides students with foundational knowledge and stimulates entrepreneurial thinking (Gibb et al., 2012). So who can be educated to be an entrepreneur easier? Entrepreneurs are the foundation of a country's economy. To make a country's economy strong, the focus should be on the young generation (Hameed and Irfan, 2019). College students with the highest potential for innovation and entrepreneurship are expected, as they have the ability to learn independently, and cultivating the spirit of innovation and entrepreneurship is easy, college students can become involved in innovation and entrepreneurial activities easier. Students who receive entrepreneurship education have higher entrepreneurial intentions (Walter and Block, 2016).

With the development of society, more and more countries have realized the importance of entrepreneurship education. There is growing interest in how education can enhance entrepreneurship by encouraging innovation (Fayolle and Gailly, 2015). The Chinese government dvocates “mass entrepreneurship and innovation” and vigorously promote entrepreneurial policies to encourage college students to start businesses. To respond to national policies, universities have successively established entrepreneurship education programs, which are crucial in promoting students' self-employment that improves entrepreneurial competence and enhances entrepreneurial intention. According to the Employment Survey Report of Chinese College Students by the Max Research Institute, the rate at which college students were starting businesses 6 months after graduation was higher in the Pan-Yangtze River Delta region than in other regions from 2014 to 2016. Furthermore, college students start businesses more vigorously, which has certain research value. The Outline of the Regional Integration Development Plan for the Yangtze River Delta, issued by the Central Committee and the State Council in December 2019, states that, “jointly creating a favorable environment for employment and entrepreneurship. we will implement targeted programs and plans to help key groups such as college graduates, migrant workers and ex-servicemen find jobs and start their own businesses.” Therefore, to explore the mechanisms and factors influencing the self-employment of college graduates in the Yangtze River Delta region with strong innovation vitality has particular practical value and provides Chinese experience as an example for other countries, especially developing countries.

Entrepreneurship education has developed rapidly since first proposed. Current studies confirm that entrepreneurship education actively promotes entrepreneurial intention (Piperopoulos and Dimov, 2015; Walter and Block, 2016) and improves entrepreneurial competence (Jiang et al., 2017; Byun et al., 2018). Some scholars have suggested that entrepreneurship education is the main driving force for improving the development of entrepreneurial ability (Draycott and Rae, 2011) and believe that entrepreneurship education can influence and improve entrepreneurial competence. However, few studies consider the mediating role of entrepreneurial competence in entrepreneurial education and entrepreneurial intention. Although some scholars have suggested that business plan competitions and entrepreneurial practice projects can improve entrepreneurial ability, stimulate entrepreneurial consciousness, and enhance entrepreneurial willingness (Zhang et al., 2014), this is supported through theoretical construction and has not been proven by experience. Therefore, based on 5,603 samples in this study, we explore the specific influence path of entrepreneurial education and entrepreneurial competence on entrepreneurial intention. This study analyzes the influence of entrepreneurship education on entrepreneurial intention from the perspective of planned behavior theory. From discussing the role of entrepreneurship education in colleges and universities on economic and social development, a conceptual model was established. This study proposes hypotheses to understand the relationship between entrepreneurial ability as an intermediary and entrepreneurial intention, and we provide evidence, in the Chinese context, for the relationship between entrepreneurial education and entrepreneurial intention.

The contents of this study include four aspects. First, based on behavioral planning theory, we review the literature and propose hypotheses for testing. Second, we use regression analysis based on the data to verify the hypotheses. Third, we analyze and discuss the results of the empirical analysis and propose theoretical contributions and practical significance based on the results. Fourth, we summarize the study, along with limitations, and suggest future research opportunities.

## Theoretical Background And Hypothesis Development

### Theory of Planned Behavior

To better understand entrepreneurial intention, this study is based on the framework of the theory of planned behavior. The theory of planned behavior is a psychological theory that focuses on attitudes, subjective norms, and perceived behavior control, which help us understand the intention of individual behaviors (Ajzen, 1991). Ajzen (2005) proposes that, when the likelihood of success is high, individuals will pay more attention to their intentions.

In the context of entrepreneurship education, this theory is helpful in analyzing the process of entrepreneurial behavior. As the goal of entrepreneurship education is not necessarily that all participants start a business in the short term, we do not use entrepreneurial behavior as a predictor. To promote entrepreneurship among college students, it is necessary to ensure that college students have high entrepreneurial intention. This study employs the premise of the theory of planned behavior to use intention as a substitute for behavior, which is a relevant tool for understanding the development of entrepreneurial intention through entrepreneurship education. Since the acquisition of knowledge can change behavior, entrepreneurial intent can be influenced by learning outcomes. Learning different behaviors that change attitudes will affect entrepreneurial intentions (Ferreira et al., 2017). Entrepreneurship education can change an individual's ability (including entrepreneurial knowledge, skills, and spirit) to change the intentions related to entrepreneurship. Particularly among young people, education influences attitudes toward entrepreneurship (Batanero et al., 2016).

### Effect of Entrepreneurship Education on Entrepreneurial Intention in Universities

Entrepreneurship education emerged abroad in the 1980s and the 1990s. In 1989, the United Nations Educational, Scientific, and Cultural Organization (UNESCO) held an international symposium on Education for the twenty-first century, in which Colin put forward the concept of entrepreneurship education, namely, career ambition and pioneering skills education. Entrepreneurship is a comprehensive concept that is not only a mere creation business, but also a way of life and thinking for students (Kuratko, 2005). It can be understood that entrepreneurship education not only means teaching students to set up enterprises, but also, more importantly, cultivating students' initiative and innovation. John Dewey, an American pragmatic educator, in his book Democracy and Education written in 1916, divided the types of entrepreneurial education courses into disciplinary courses and activity courses. Ginanjar (2016) defines entrepreneurship education as an entrepreneurship education course offered by universities that teaches the theory and practice of entrepreneurship. Benson (2004) discussed teaching methods, compared simulation and contingency-based empirical theory, and proposed that entrepreneurship education should form a system with its own characteristics instead of simply introducing standard and conventional teaching methods.

Teaching methods for entrepreneurship education are not static, but dynamic, and may change with the constant use of social media as part of the learning experience (Chawinga, 2017). Ratten and Usmanij (2021) argue that entrepreneurship education is an experiential learning that needs to be embedded with key learning objectives in the curriculum to increase student engagement, and that a hybrid learning approach such as case studies and business plan competitions is needed. Neck and Greene (2011) believe that entrepreneurship is a way of thinking and behaving, and entrepreneurship courses, different from other theoretical courses, need to apply practice in course content, such as carrying out competitions, simulating entrepreneurship, and reflective practice. On this basis, entrepreneurship education in universities is divided into three dimensions: entrepreneurial teaching, business plan competition, and entrepreneurial practice support.

Intention is considered to be an important factor in choosing a future career (Franco et al., 2010). Especially when it comes to explaining a decision to start a new business, entrepreneurial intent plays an important role, a mindset that directs people's attention or action toward a particular behavior (Ferreira et al., 2012). Shapero and Sokol (1982) argue that intentions are determined by feasibility, intentionality, and the propensity to act. According to the theory of planned behavior, a person's entrepreneurial intention is a function of one's positive attitude, favorable subjective norms, and positive perceived behavioral control for entrepreneurial behavior. According to Ginanjar (2016) the purpose of entrepreneurship education is to equip students with the ability to understand and practice entrepreneurship, and causes changes in attitudes, norms, and behaviors.

Entrepreneurship education is the process of teaching students entrepreneurial, which involves identifying viable business opportunities and turn them into successful commercial ventures (Matlay et al., 2012). Entrepreneurship education plays a key role in cultivating students' intentions (Anwar and Saleem, 2019). Empirical research by Kaya et al. (2019) proves that both entrepreneurial support and self-management-related skill teaching increase the possibility of future entrepreneurial activities, which reinforces the necessity of establishing entrepreneurship education courses in universities. Shirokova et al. (2017) empirically conclude that courses, extracurricular activities, and financial support related to University entrepreneurship have different effects on the propensity of experienced and inexperienced entrepreneurs and that these effects influence each other and are carried out simultaneously. Morris et al. (2017), applying the concept of embeddedness, assesses the influence of the University entrepreneurship environment on college students' entrepreneurial activities. The results show (1) a positive correlation between college students who participated in courses related to entrepreneurship and participation in entrepreneurial activity; (2) that extracurricular activities and student University funding were negatively correlated with entrepreneurial activity; and (3) a negative relationship between financial support and entrepreneurial activity. On the basis of the theory of planned behavior.

First, entrepreneurship teaching is the most basic aspect of entrepreneurship education. When students had little knowledge about entrepreneurship, entrepreneurship courses had a significant effect on their entrepreneurial intention. A randomized study of 3,775 students who graduated from 1985 to 2009 found that taking two or more entrepreneurship courses had a positive impact on students' entrepreneurial intentions and their ability to become actual entrepreneurs, both at graduation and well after graduation (10 years or more). However, some scholars have found that, although entrepreneurship teaching improves students' knowledge and ability, students' entrepreneurial intention is not necessarily improved in this way.

Second, entrepreneurship is a complex social activity that requires a higher practical ability. The effectiveness and value of experiential learning is practically beyond debate in entrepreneurial education (Mandel and Noyes, 2016). Gibb (2011) argues that development of the entrepreneurial mindset requires learning by doing, conversion of knowledge into problem-solving methods. As an aspect of experiential learning, business plan competition can let students know the real situation of entrepreneurship and promote entrepreneurial intention. However, current entrepreneurship competitions are formalized, and increasingly, organizers and participants are more concerned with the arena, scale, and number of prizes, which increases the gap between the entrepreneurial process perceived by students and the real-world entrepreneurial situation (Chun-Yan et al., 2017). After students have a clear and practical understanding of the preparation required for real entrepreneurship, they lose overoptimistic attitudes toward entrepreneurship and interests dampen (Oosterbeek et al., 2010).

Third, Entrepreneurial practice support is a very important incentive measure. In the early stage of entrepreneurship, students are in lack of space, resources and funds, etc., so government and schools should provide support, such as setting up venture funds to provide certain financial support for entrepreneurial students, or even providing free space to help students find resources (Zhao and Zhao, 2021). This will increase the success rate of entrepreneurship and make students willing to start business. In addition, professional guidance on entrepreneurship reduces the obstacles for students to start a business, making it easier to start a business.

This study proposes the following:

**Hypothesis 1 (H1)**. *Entrepreneurship education in universities has a positive impact on entrepreneurial intention*.
**Hypothesis 1a (H1a)**. *Entrepreneurial teaching has a positive impact on entrepreneurial intention*.
**Hypothesis 1b (H1b)**. *Business plan competitions have a positive impact on entrepreneurial intention*.
**Hypothesis 1c (H1c)**. *Entrepreneurial practice support has a positive impact on entrepreneurial intention*.

### Mediating Effect of Entrepreneurial Competence on the Influence Mechanism of Entrepreneurship Education on Entrepreneurial Intention in Universities

Scholars agree that entrepreneurship is a key driver of the economy. Uku and Marge (2017) argued that society is improved not only by entrepreneurship but by entrepreneurial individuals with knowledge, attitudes, and skills to identify and exploit opportunities, create value, and orient toward action. Generally, competence is the ability to successfully solve problems of reality, challenges, and opportunities (Barth et al., 2007). In the last decade, core competence has become a pillar of education development in the European Union. The March 2002 progress report defined core competencies as “representing a set of knowledge, skills, and attitudes.” As part of the core competencies, entrepreneurial competence is defined as “the ability to take action on opportunities and ideas and turn them into value for others.” According to Pittaway and Cope (2007), entrepreneurial competence includes initiative, creativity, innovation, and the ability to take risks, as well as the ability to plan and manage projects. In the face of increasing complexity and uncertainty, entrepreneurial competence provides the attitude, skills, and knowledge that enable an individual to identify opportunities, solve problems, and develop them sustainably. Therefore, this study argues that the entrepreneurial competence of college students includes both entrepreneurial skills and entrepreneurial knowledge and spirit.

A review of the literature reveals that entrepreneurial ability is influenced by learning and educational processes. Rideout and Gray (2013) believe that entrepreneurship education in universities is a comprehensive and practical educational activity that imparts entrepreneurial knowledge and trains students' entrepreneurial consciousness, strategic choice, opportunity identification, and other abilities. Liñán et al. (2011) emphasizes that entrepreneurship education has been considered a key instrument for increasing the entrepreneurial attitudes of potential and nascent entrepreneurs. The essence of entrepreneurship education is to cultivate students' entrepreneurial consciousness, thinking, and skills (Jones and English, 2004). Solesvik (2013) believe that entrepreneurship education promotes the improvement of students' entrepreneurial competence. However, most entrepreneurship education professors focus on entrepreneurship, and students learn to understand entrepreneurship mainly from theoretical perspectives (Lackéus, 2015). From a narrowly defined perspective, students are supported as entrepreneurs. From a broad perspective, entrepreneurship education encourages students to be innovative and proactive, enabling them to acquire entrepreneurial ability and take entrepreneurial actions. Since the narrow definition of entrepreneurship education is commonly used, the success of entrepreneurship education is generally measured by the intention of students or the number of students who start businesses (Lackéus, 2015). Such studies do not explain what role entrepreneurial competence has on entrepreneurial education and entrepreneurial intention. However, entrepreneurship education based on the cultivation of entrepreneurial ability has a crucial influence on whether students start a business (Uku and Marge, 2017).

Entrepreneurship education teaches theoretical knowledge and shares successful experiences, which cultivate students' entrepreneurial competence and growth. However, the business plan competition methodology can cultivate the ability of entrepreneurs and is a valuable learning experience in the pursuit of new skills. Furthermore, it can motivate participants to improve abilities and self-confidence (Watson et al., 2018). The cultivation of practical ability is essential in improving students' comprehensive competence and fostering an innovative spirit. Students participating in entrepreneurship competitions obtain direct experience, making entrepreneurship projects easier in the future. A school's entrepreneurship practice provides office space, facilities, and equipment, as well as venture funds, so that students' ideas can flourish, and entrepreneurship can be realized. Using a cross-sectional sample of 496 German scientists, Obschonka et al. (2010) finds a correlation between early entrepreneurial ability and entrepreneurial intention. Applying the structural equation model to study the influence of college students' entrepreneurial intention, Peng et al. (2012) verify that entrepreneurial attitude, entrepreneurial self-efficacy, and entrepreneurial competence have significant direct or indirect influences on entrepreneurial intention. Through empirical tests, Hu and Xu (2015) concludes that entrepreneurial attitude and knowledge have a significant impact on entrepreneurial intention, while entrepreneurial competence does not have a significant impact on entrepreneurial intention. Therefore, this study proposes the following:

**Hypothesis 2 (H2)**. *Entrepreneurial competence has a mediating effect on the mechanism by which entrepreneurship education in universities influences entrepreneurial intention*.
**Hypothesis 2a (H2a)**. *Entrepreneurial competence has a mediating effect on the influence mechanism of entrepreneurial teaching on entrepreneurial intention*.
**Hypothesis 2b (H2b)**. *Entrepreneurial competence mediates the influence of business plan competition on entrepreneurial intention*.
**Hypothesis 2c (H2c)**. *Entrepreneurial competence has a mediating effect on the influence of entrepreneurial practice support on entrepreneurial intention*.

**Figure 1:**
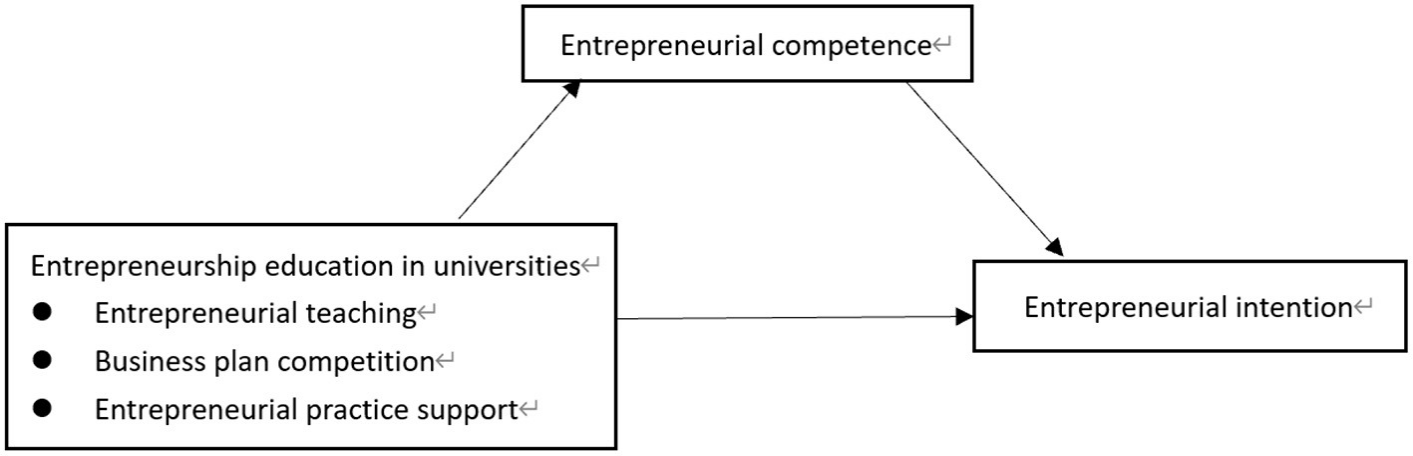
Theoretical model.

Figure 1 illustrates the theoretical model of this study.

## Research Methods And Data Analysis

### Sample and Procedure

This study uses data from a 2017 to 2018 questionnaire survey conducted by the China Innovation and Entrepreneurship Education Research Institute for undergraduate students (except freshmen from 2018). The questionnaire uses a comprehensive design based on the literature of domestic and foreign periodicals, a comparative analysis of various existing questionnaires on innovation and entrepreneurship education, and an in-depth semi-structured interview analysis by several experienced teachers of entrepreneurship education.

Data were collected from students through alumni WeChat groups, student classes, and emails in the Yangtze River Delta urban agglomeration. We collected 5,603 effective questionnaires from students with practical experience. Respondents included individuals from 13 majors; of these 46.2% were female and 53.8% male, and 92.84% were from non-double-top universities. College students with practical experience can more accurately understand the impact of entrepreneurship teaching, business competition, and entrepreneurial practice support on their entrepreneurial intentions, which enhances the accuracy of this study. The basic information of the sample is shown in Table 1.

**Table 1:** Descriptive statistics (*N* = 5,603).

**Demographic variables**	**Dimension**	**Frequency**	**Percentage (%)**
Gender	Male	3,017	53.80
	Female	2,586	46.20
Major	Philosophy	56	1.00
	Economics	1,331	23.80
	Science of Law	86	1.50
	Pedagogy	156	2.80
	Literature	216	3.90
	History	17	0.30
	Science	486	8.70
	Engineering	1,212	21.60
	Agriculture	252	4.50
	Medical Science	331	5.90
	Military Science	19	0.30
	Management	997	17.80
	Art	444	7.90
Education resources [Type of school you are attending (“Double First-Class” University)]	Yes	401	7.16
	No	5,202	92.84

### Measures

Entrepreneurship teaching follows the questionnaire developed by Hosseini and Pouratashi (2011) and Wiley and Berry (2015), and we measure three indicators on a 5-point scale: “There are various types of entrepreneurship education courses,” “teachers have entrepreneurial experience,” and “the contents of entrepreneurship courses are closely combined with their professional knowledge” (1 = strongly disagree; 5 = strongly agree). The reliability of α is 0.898.

The business plan competition follows the content of Bolli and Woerter (2013) and Falck and Woessmann (2013), and we measure three indicators on a 5-point scale: “Business plan competitions improve entrepreneurial confidence,” “business plan competitions expand interpersonal networks,” and “business plan competition improves teamwork ability” (1 = strongly disagree; 5 = strongly agree). The reliability of α is 0.938.

Entrepreneurship practice support follows Greefs (1998), Zou and Zhao (2014), and Huang et al. (2020) and we measure four indicators on a 5-point scale: “Entrepreneurial practice is supported by a special entrepreneurial fund,” “the school provides integrated entrepreneurial practice services,” “entrepreneurial practice has a special off-campus practice base,” and “entrepreneurial practice projects are highly integrated with professional learning” (1 = strongly disagree; 5 = strongly agree). The reliability of α is 0.943.

Entrepreneurial competence follows Uku and Marge (2017) and Pittaway and Cope (2007), and we measure three indicators on a 5-point scale: “Entrepreneurship education helps to enrich entrepreneurial knowledge,” “entrepreneurship education helps to cultivate innovative spirit,” and “entrepreneurship education helps to improve entrepreneurial skills” (1 = strongly disagree; 5 = strongly agree). The reliability of α is 0.958.

Indicators of entrepreneurial intention follow Díaz-García and Jiménez-Moreno (2010) and Zhang et al. (2014), and we measure one indicator on a 5-point scale: “I will start a business in the next year” (1 = strongly disagree; 5 = strongly agree).

In selecting control variables, studies have found that graduates of social sciences, natural sciences, medicine, and education from prestigious universities are significantly different from graduates of other universities in their choice of entrepreneurship (Daghbashyan and Hârsman, 2014). Research has found that the entrepreneurial intention of science and engineering universities has a significant impact on entrepreneurship education (Souitaris et al., 2007; Zhang et al., 2014), and scholars have studied the influence of MBA courses on students' entrepreneurial intention (Zhao et al., 2005). Therefore, we select double first-class universities, medicine, education, science, engineering, management, and economics as control variables.

### Reliability and Validity Test

First, we tested overall reliability and validity. The α-value of the scale was 0.943, the Kaiser-Meyer-Olkin (KMO) value was 0.930, the α-value was ≥0.7, and the KMO-value was >0.5, indicating good reliability and validity of the overall scale. Second, we tested the reliability and validity of each factor. The results in Table 2 show that all factors passed the internal consistency test (α-value ≥0.7), indicating that the reliability of each factor scale is good. The KMO and Bartlett sphere test results of each factor show that all variables pass the Bartlett sphere test (KMO-value >0.5), which met the factor analysis standard. The results of the exploratory factor analysis show that the factor load of each item after rotation was >0.6, the combined reliability (CR) of all factors was >0.7, and the Average variance extracted (AVE) value of each factor was >0.5, indicating good convergent validity of the scale. Third, we calculated the square roots and correlation coefficients of AVE-values of each factor (Table 3), and the results show that each factor scale had good discriminant validity.

**Table 2:** Reliability and validity of each factor.

**Factor**	**Measuring item**	**Factor loading**	**Percentage of interpreted variance (%)**	**α**	**KMO**	**CR**	**AVE**
ET	ET1	0.873	83.14	0.898	0.752	0.899	0.747
	ET2	0.860					
	ET3	0.860					
BPC	BPC1	0.971	88.94	0.938	0.768	0.938	0.834
	BPC2	0.919					
	BPC3	0.907					
EPS	EPS1	0.869	85.39	0.943	0.863	0.943	0.806
	EPS2	0.924					
	EPS3	0.891					
	EPS4	0.906					
EC	EC1	0.937	92.23	0.958	0.779	0.958	0.883
	EC2	0.939					
	EC3	0.943					
EI	EI	—	—	—	—	—	—

*CR, Composite reliability; AVE, Average variance extracted*.

**Table 3:** Correlation coefficient matrix between the square root of the AVE-value and factors.

	**Mean value**	**Standard deviation**	**ET**	**IBPC**	**EPS**	**EC**
ET	3.70	0.93	0.912			
BPC	3.95	0.86	0.783^**^	0.943		
EPS	3.82	0.91	0.827^**^	0.839^**^	0.924	
EC	4.00	0.86	0.716^**^	0.833^**^	0.805^**^	0.960
EI	4.01	0.89	0.692^**^	0.804^**^	0.776^**^	0.917^**^

*The values shown on the diagonal are the square root values of AVE for each factor*.

** *p < 0.01*.

### Common Method Deviation Test and Multicollinearity Test

This study used the Harman single factor test to test the deviation of common methods. The results show that, without rotation, the first factor explains 49.80% of the variation in all items, which is <50%. The results show that the degree of fit was not good (RMSEA = 0.179, CFI = 0.857, GFL = 0.643, AGFI = 0.513, IFI = 0.857, NFI = 0.857), which indicates that the problem of homology method of data used in this study was controlled. The fitting degree of the five-factor model used in this study was good (RMSEA = 0.055, CFI = 0.988, GFL = 0.969, AGFI = 0.952, IFI = 0.988, NFI = 0.987), and the common variance test (CMV) found that the fitting statistics of the model did not change significantly (RMSEA = 0.054, CFI = 0.989, GFL = 0.972, AGFI = 0.954, IFI = 0.989, NFI = 0.989). The homology method of data used in this study is controlled and the model used has good fit degree. In addition, the variance inflation factor values (VIF) among the factors are all <10, indicating no serious multicollinearity problem.

### Hypothesis Testing

#### Descriptive Statistics and Correlation Analysis of Each Variable

Correlation analysis was performed between the total average scores of ET, BPC, EPS, EC, and EI. The results (Table 3) show that EI was significantly positively correlated with ET, BPC, EPS, and EC, which, in turn, was positively correlated with ET, BPC, and EPS, and the hypothesis was preliminarily verified.

#### Mediation Model Testing

This study sets ET, BPC, and EPS as independent variables; EC as an intermediary variable; EI as the dependent variable; and double first-class universities medicine, education, science, engineering, management, and economics as control variables. This study uses the inspection theory put forward by the above model, and the results are shown in Tables 4–6. This study uses the bootstrap of SPSS macro software, and PROCESS software. We tested the mediating effect by repeating the sampling 5,000 times and calculating at the 95% confidence interval (Jie et al., 2012).

**Table 4:** The mediating effect of ET as independent variable was analyzed.

	**M: EC**			**Y: EI**		
	**coeff**	**se**	**95%CI**	**coeff**	**se**	**95%CI**
Constant	1.5217^**^	0.0367	1.4497, 1.5937	0.1787^**^	0.0246	0.1306, 0.2269
X1:ET	0.6641^**^	0.0087	0.6471, 0.6811	0.0707^**^	0.0073	0.0565, 0.0850
M:EC				0.8913^**^	0.0078	0.8759, 0.9066
Neo-confucianism	−0.0099	0.0328	−0.0742, 0.0545	0.0087	0.0192	−0.0290, 0.0463
Engineering	0.0411	0.0251	−0.0080, 0.0903	0.0026	0.0147	−0.0261, 0.314
Economics	0.0072	0.0248	−0.0414, 0.0557	−0.0085	0.0145	−0.0369, 0.0199
Management	0.0163	0.0264	−0.0354, 0.0679	0.0147	0.0154	−0.0156, 0.0449
Pedagogy	−0.0024	0.0514	−0.1031, 0.0983	−0.0034	0.0301	−0.0623, 0.0556
Medical	0.0632	0.0377	−0.0107, 0.1372	0.0136	0.0221	−0.0297, 0.0569
Education resources	0.0680^*^	0.0314	0.0063, 0.1296	0.0186	0.0184	−0.0175, 0.0547
*R*-sq		0.5129			0.8432	
*F*		736.4074^**^			3,341.6382^**^	
X1: ET → M: EC → Y: EI						
**Indicators**	**Effect**		**BootSE**	**BootLLCI**		**BootULCI**
Total effect	0.6626		0.0092	0.6445		0.6808
Direct effect	0.0707		0.0073	0.0565		0.085
Indirect effect	0.5919		0.0116	0.5698		0.6146

* *p < 0.05*,

** *p < 0.01*.

Table 4 shows that X1 entrepreneurship teaching can significantly positively influence M: entrepreneurial competence (A_1x1_ = 0.6641, 95%CI = 0.6471 – 0.6811), and M: entrepreneurial competence can also significantly positively influence Y: entrepreneurial intention (B_1x1_ = 0.8913, 95%CI = 0.8759–0.9066). When M: entrepreneurial competence is added into the model, X1: entrepreneurial teaching can significantly positively influence Y: entrepreneurial intention (C '_1X1_ = 0.0707, 95%CI = 0.0565–0.0850). Hence, M: entrepreneurial competence plays a partial intermediary role between X1: entrepreneurial teaching and Y: entrepreneurial intention.

Table 5 shows that X2 business plan competition can significantly positively influence M: entrepreneurial competence (A_1X2_ = 0.8276, 95%CI = 0.8132 – 0.8421), and M: entrepreneurial competence can significantly positively influence Y: entrepreneurial intention (B_1X2_ = 0.8331, 95%CI = 0.8139 – 0.8523). When M: entrepreneurial competence is added into the model, X2 business plan competition has a significantly positive influence on Y: entrepreneurial intention (C '_1X2_ = 0.1345, 95%CI = 0.1154 – 0.1536). Hence, M: entrepreneurial competence plays a partial mediating role between X2 business plan competition and Y: entrepreneurial intention.

**Table 5:** Moderated mediating effect analysis with BPC as independent variable.

	**M: EC**			**Y: EI**		
	**coeff**	**se**	**95%CI**	**coeff**	**se**	**95%CI**
Constant	0.7256^**^	0.0322	0.6625, 0.7887	0.1431^**^	0.0246	0.0948, 0.1914
X2:BPC	0.8276^**^	0.0074	0.8132, 0.8421	0.1345^**^	0.0097	0.1154, 0.1536
M:EC				0.8331^**^	0.0098	0.8139, 0.8523
Neo-confucianism	0.0282	0.0260	−0.0228, 0.0792	0.0144	0.0191	−0.0229, 0.0518
Engineering	0.0046	0.0199	−0.0344, 0.0435	−0.0008	0.0146	−0.0294, 0.0277
Economics	0.0179	0.0196	−0.0205, 0.0563	−0.0061	0.0144	−0.0343, 0.0220
Management	−0.0050	0.0209	−0.0459, 0.0359	0.0122	0.0153	−0.0177, 0.0422
Pedagogy	0.0432	0.0407	−0.0366, 0.1230	0.0037	0.0298	−0.0547, 0.0622
Medical	−0.0349	0.0299	−0.0936, 0.0237	0.0013	0.0219	−0.0416, 0.0442
Education resources	−0.0172	0.0249	−0.0661, 0.0316	0.0085	0.0182	−0.0272, 0.0443
*R*-sq		0.6941			0.8458	
*F*		1,586.6214^**^			3,408.7211^**^	
X2: BPC → M: EC → Y: EI						
**Indicators**	**Effect**		**BootSE**	**BootLLCI**		**BootULCI**		
Total effect	0.8240		0.0082	0.8080		0.8400		
Direct effect	0.1345		0.0097	0.1154		0.1536		
Indirect effect	0.6895		0.0134	0.6635		0.7162		

** *p < 0.01*.

Table 6 shows that X3 entrepreneurial practice support can significantly positively influence M: entrepreneurial competence (a1X3 = 0.7624, 95%CI = 0.7476 – 0.7771), and M: entrepreneurial competence can significantly positively influence Y: entrepreneurial intention (b_1X3_ 0.8557, 95%CI = 0.8377 – 0.8737). When M: entrepreneurial competence is added into the model, X3 entrepreneurial practice support has a significantly positive influence on Y: entrepreneurial intention (c '_1X3_ = 0.1059, 95%CI = 0.0889 – 0.1229). Hence, M: entrepreneurial competence plays a partial mediating role between X3 entrepreneurial practice support and Y: entrepreneurial intention.

**Table 6:** Moderated mediating effect analysis with EPS as independent variable.

	**M: EC**			**Y: EI**		
	**coeff**	**se**	**95%CI**	**coeff**	**se**	**95%CI**
Constant	1.0544^**^	0.0326	0.9906, 1.1182	0.1755^**^	0.0244	0.1278, 0.2233
X3:EPS	0.7624^**^	0.0075	0.7476, 0.7771	0.1059^**^	0.0087	0.0889, 0.1229
M:EC				0.8557^**^	0.0092	0.8377, 0.8737
Neo-confucianism	0.0174	0.0279	−0.0372, 0.0720	0.0123	0.0191	−0.0252, 0.0498
Engineering	0.0411	0.0213	−0.0006, 0.0828	0.0042	0.0146	−0.0244, 0.0328
Economics	0.0335	0.0210	−0.0077, 0.0746	−0.0043	0.0144	−0.0325, 0.0240
Management	0.0674^**^	0.0224	0.0235, 0.1112	0.0225	0.0154	−0.0077, 0.0526
Pedagogy	−0.0039	0.0436	−0.0893, 0.0816	−0.0039	0.0299	−0.0626, 0.0548
Medical	0.0968^**^	0.0320	0.0341, 0.1596	0.0205	0.0220	−0.0227, 0.0636
Education resources	−0.0069	0.0267	−0.0592, 0.0454	0.0104	0.0183	−0.0255, 0.0463
*R*-sq		0.6491			0.8447	
*F*		1,293.6873^**^			3,379.1420^**^	
X3: EPS → M: EC → Y: EI						
**Indicators**	**Effect**		**BootSE**	**BootLLCI**		**BootULCI**		
Total effect	0.7583		0.0082	0.7421		0.7744		
Direct effect	0.1059		0.0087	0.0889		0.1229		
Indirect effect	0.6523		0.0125	0.6282		0.6774		

^*^p < 0.05,

** *p < 0.01*,

^***^p < 0.001.

## Discussion

This study analyzes the influence of three aspects of entrepreneurship education on entrepreneurial intention and reveals the mediating role of entrepreneurial competence in this process. The main conclusions include the following. Entrepreneurship teaching, business plan competition, and entrepreneurial practice support have a positive impact on entrepreneurial intention. Entrepreneurial competence plays an intermediary role in entrepreneurship teaching, business plan competition, entrepreneurship practice support, and entrepreneurship intention.

### Theoretical Implications

First of all, the theoretical significance of this study is to confirm the three elements of entrepreneurship education, respectively: relationship between entrepreneurial teaching, business plan competition, entrepreneurial practice support, and entrepreneurial intention. The results show that entrepreneurship teachers' guidance, entrepreneurial practical support, and students' participation in business plan competition can improve the entrepreneurial intention of universities. This is contrary to the research conclusion of Chen et al. (2015): “Students who have received entrepreneurship education really understand that starting a business is not an easy thing, so entrepreneurship has not been improved immediately.” Although entrepreneurship is an activity of high risk and high uncertainty, entrepreneurship education can improve the knowledge and ability of potential entrepreneurs, increase their entrepreneurial self-efficacy, and thus improve their entrepreneurial intention.

Secondly, the mediation model of entrepreneurial education, entrepreneurial competence and entrepreneurial intention is established. The results show that entrepreneurship education can not only directly promote the entrepreneurial intention of college students, but also indirectly promote the entrepreneurial competence of college students. This paper clarifies the role of entrepreneurial education in improving college students' entrepreneurial intention by improving their entrepreneurial competence, and emphasizes the partial mediating role of entrepreneurial competence. This model not only improves the theory of the relationship between entrepreneurial intention and antecedent variables, but also can be used to evaluate the impact of entrepreneurial education on entrepreneurial intention and career development of Chinese college students. It has a certain theoretical guiding significance for college students to promote entrepreneurship education.

Finally, this study is based on the theory of planned behavior and develops the theory of planned behavior. The conclusions of this study extend the work of Fayolle and Gailly (2015), who show that entrepreneurship education programs reduce students' intentions to initially consider an entrepreneurial career. This study finds that entrepreneurship education promotes the entrepreneurial intention of students with practical experience. This study provides new insights regarding the role of entrepreneurship education and entrepreneurial intentions of college students. This study and model can be used to assess the impact of entrepreneurial teaching, business plan competition, and entrepreneurial practice support on entrepreneurial intention among college students.

### Practical Implications

Regarding the entrepreneurial intention of college students, entrepreneurial education should aim to improve entrepreneurial competence, and even if students have no intention of starting a business at present, entrepreneurial education can lay a foundation for the future. Luis-Rico et al. (2020) shows that the cultivation of entrepreneurial competence has an impact on entrepreneurial intention, and education and curriculum reform must promote the development of entrepreneurial ability at all stages of education to improve entrepreneurial intention. Some scholars find that the ability to start a business in early adolescence has a positive impact on the entrepreneurial process and can promote multiple cases of entrepreneurship for an individual's career (Obschonka et al., 2011). Therefore, strengthening entrepreneurship education and cultivating entrepreneurial competence encourages college students to start businesses in the future.

First, entrepreneurship teaching can be divided into teachers and courses. Regarding teachers, universities should consider the development of entrepreneurship teachers. In particular, they should strengthen innovation and entrepreneurship training for tutors and other student teachers. Some incentives could be adopted. Universities should actively recruit entrepreneurs or successful graduates to hold special lectures and courses on a regular basis to better guide entrepreneurial practices for students. Regarding courses, some scholars suggest enacting methodological and strategic changes in education, such as avoiding excessive use of theory-based methods, encouraging action-based learning and direct participation, and developing independent learning to promote the development of entrepreneurial competence (Arranz et al., 2017). Universities should refine curricula to build a complete system of entrepreneurship education courses.

Second, the majority of students agree that business plan competitions are helpful. Through the business plan competition, college students learn entrepreneurial knowledge, obtain entrepreneurial practice, enhance entrepreneurial confidence, and strengthen entrepreneurial spirit. Simultaneously, participation in competitions can expand interpersonal relationships and encourage like-minded business partnerships. The focus and purpose of the competitions should be to help participants obtain the required skills, knowledge, motivation, and funding resources to solve complex issues involved in business. Through this, they are able to learn how to create and maintain enterprises. Therefore, the design of this type of competition requires further optimization. For example, business plan competitions should ensure that all participants provide timely feedback to assess the weak areas of business plans for improvement, which will help them both in the competition and perhaps to continue the business in the future (Cant, 2018). Business plan competitions should provide a reasonable competition mode as well as have a fair platform. Furthermore, the participation and support of various sectors of society should be paramount.

Third, in terms of entrepreneurial practice support, an increase in convenient access to support and services provided by colleges and universities, such as capital, locations, guidance, and training, increases the number of students who will develop an understanding of entrepreneurial activities and decrease worries about entrepreneurship in the future. This conclusion is similar to De-peng and Bao-shan (2017), where tax incentives, the entrepreneurial environment, and supporting measures have a positive impact on entrepreneurial intention. Therefore, universities should strengthen cooperation between the government and enterprises. Furthermore, the support provided by the government can help a start-up enterprise survive, which will attract more college students to join the entrepreneurial team.

### Limitations and Future Research Recommendations

First, this study adopted a cross-sectional design, and the data only represents the situation of college students' entrepreneurship education during a certain time. Additionally, the study only selected the topics of entrepreneurship education for college students. Most of the research variables are subjective evaluations; therefore, it is difficult to completely avoid the deviation caused by subjective opinions. Future research can expand entrepreneurship education in more aspects, which can be discussed from a global perspective. Additionally, future research could track the relationship between variables involved in some entrepreneurs at different time points, conduct research on gender differentiation, or expand the study the optimization of entrepreneurship education for college students.

Second, existing studies on the influence of entrepreneurship education on entrepreneurial intention are based on the development of already developed Western countries. Entrepreneurial educators should adjust curricula in view of cultural differences in learning (Bandera et al., 2018). The growing emphasis on entrepreneurship education in Asia, especially China, and the increasing number of students participating in business plan competitions allow students to focus on skills that can be applied to business practices in home countries. Considering the particularity of culture and society, the implementation of entrepreneurship education should combine local culture and national conditions.

Finally, the current social and business environment is dynamic, and with the progress of technology, the way people teach and learn changes. Entrepreneurship education should incorporate innovative technologies, such as digital technology and AI. Within an existing digital ecosystem, education should provide more free online courses and services on educational issues (Gunkel, 2017). The role of social media in entrepreneurship courses is noteworthy (Wu and Song, 2019). These innovative technologies and models can provide greater space for entrepreneurship education in the future.

## Conclusion

Entrepreneurship education has a significant impact on entrepreneurial intention. To promote economic development, more countries have introduced policies and measures to strengthen entrepreneurship education, among which entrepreneurship education in universities is vital. However, society is improved by both entrepreneurship and individuals with entrepreneurial ability. Scholars and practitioners should understand entrepreneurship beyond the narrow sense (starting a business) into the broad sense (recognizing and solving problems, identifying opportunities, and creating value in society). Therefore, entrepreneurial competence is an essential focus. Hence, we can continue to create value for society, continue entrepreneurship, and promote economic development in an increasingly complex and uncertain society.

## Data Availability Statement

The original contributions presented in the study are included in the article/supplementary material, further inquiries can be directed to the corresponding author/s.

## Ethics Statement

Ethical review and approval was not required for the study on human participants in accordance with the local legislation and institutional requirements. Written informed consent from the (patients/participants or patients/participants legal guardian/next of kin) was not required to participate in this study in accordance with the national legislation and the institutional requirements.

## Author Contributions

YH and YC described and developed the review and the hypothesis. YH, YL, LH, JW, LA, XH, and TC was involved in the data collection process. YH and YS performed the analysis, interpretation of the results, and formulated the main conclusions. YC and YL formulated the study limitations and future directions for research. All authors helped editing, formatting the paper, and contributed equally to this paper.

## Conflict of Interest

The authors declare that the research was conducted in the absence of any commercial or financial relationships that could be construed as a potential conflict of interest.
